# Common Ground between Biological Rhythms and Forensics

**DOI:** 10.3390/biology11071071

**Published:** 2022-07-18

**Authors:** Klara Janjić, Christoph Reisinger, Fabian Kanz

**Affiliations:** 1 University Clinic of Dentistry, Medical University of Vienna, 1090 Vienna, Austria; klara.janjic@meduniwien.ac.at; 2 Center for Forensic Medicine, Medical University of Vienna, 1090 Vienna, Austria; christoph.reisinger@meduniwien.ac.at

**Keywords:** chronobiology, forensic genetics, aging, chronobiology disorders, cause of death, forensic anthropology

## Abstract

**Simple Summary:**

Biological clocks regulate the timing of numerous body functions in adaption to daily repeating cycles in the environment, such as the sleep–wake phases that are trained by the cycling changes of night and day light. The identification of a deceased victim is a critical component in a forensic investigation, but it can be significantly hampered by the condition of the dead body and the lack of personal records and documents. This review links current knowledge on the molecular mechanisms of biological rhythms to forensically relevant aspects, including the time period since death, cause of death, the use of insects for forensics, sex and age of a person, ethnic background and development. Putting these findings in context demonstrates how the analysis of molecular clock analysis could be used as tool for future personal identification in forensic investigations.

**Abstract:**

Biological clocks set the timing for a large number of essential processes in the living human organism. After death, scientific evidence is required in forensic investigations in order to collect as much information as possible on the death circumstances and personal identifiers of the deceased victim. We summarize the associations between the molecular mechanisms of biological rhythms and forensically relevant aspects, including post-mortem interval and cause of death, entomological findings, sex, age, ethnicity and development. Given their importance during lifetime, biological rhythms could be potential tools to draw conclusions on the death circumstances and the identity of a deceased person by mechanistic investigations of the different biological clocks in a forensic context. This review puts the known effects of biological rhythms on the functions of the human organism in context with potential applications in forensic fields of interest, such as personal identification, entomology as well as the determination of the post-mortem interval and cause of death.

## 1. Introduction

Biological clocks are molecular machineries that synchronize essential biological functions to cycling environmental changes. Thus, this mechanism helps an organism to achieve an optimal adaption of behavior and functions to its natural habitat. The length period defines the type of biological rhythm, ranging from milliseconds, as in the oscillating information of neuronal networks [1], to hundreds of years, as suggested for the molecular rate of evolution across species [2]. Physical forces produce different kinds of environmental cycles, such as the periodic changes of day and night, water levels or seasons. These cues from the environment that entrain a biological rhythm are commonly known as zeitgebers within the field of chronobiology. In adaption to these cycling changes of light and dark, wet and dry as well as warm and cold and their respective length periods, organisms can generally adapt to four different types of environmental rhythms, including circatidal (12.4 h periods) [3], circadian (24 h periods) [4], circalunar (29.5 d periods) [5] and circannual (365.25 d periods) [6] rhythms. These rhythms either align with solar or with lunar geophysical effects. Circalunar rhythms seem to impact the synchronization of various fundamental behavioral features for survival, such as feeding, activity levels or migration across different species, but the precise signaling mechanisms and their molecular key elements remain unrevealed [7]. Unlike the state of knowledge on lunar rhythms, the role and importance of the circadian clock are the most prominent number of findings among all biological rhythms in the literature and the discovery of its underlying molecular mechanism was honored with the Nobel Prize in Physiology or Medicine in 2017. Its relevance in different medical fields, such as neurology [8], nephrology [9], hepatology [10], dermatology [11], pharmacology [12], oncology [13] and pathology [14], or biological aspects, such as microbial activity [15], metabolism [16], immunology [17], aging [18], genomics [19], proteomics [20] and epigenetics [21], is supported by extensive scientific evidence, which emphasizes the essential role of functioning biological rhythms in the life of an individual and the development of a whole species [22], but what happens when the clocks stop ticking?

In cases of suspicious death, the identification of the victim is a key objective of each forensic investigation. The physical appearance of a body can change considerably after natural disasters, accidents, late stages of decomposition or other external causes, turning the personal identification of human remains into a challenging task that requires time- and cost-consuming methods [23] or at least the recovery of victim samples with sufficient DNA concentrations [24]. Additionally, many of these methods depend on the availability of ante-mortem data, such as health records or genetic material to enable an adequate interpretation and contextualization for the respective case. These can be information on sex, age, physical appearance, ethnicity, lifestyle factors such as social environment, nutrition or medical history. Finally, to resolve unexplained death circumstances, evidence needs to be collected to determine time and cause of death. Findings associated with the location of found human remains can add answers to these aspects. Apart from human remains, evidence can be collected from the surrounding environment of the find spot, which can be samples from soil [25] or insects [26].

While originally biological clocks synchronize organismal functions to the natural environment, modern life is influenced by social factors rather than a natural habitat. Working hours, free time activities, housing, travelling habits, exposure to artificial light sources and similar factors compose such a social environment and shift biological rhythms. The chronotype of a person reflects the preference of daytime for certain activities and behaviors. On the scale of chronotypes, the morning and the evening type represent the two extreme types, which show a certain prevalence in correlation with different diseases such as type 2 diabetes mellitus [27], success of treatment timing such as in light therapies for breast cancer survivors [28] and habits such as snacking behavior [29], satisfaction with work schedules [30] or sleep times during pandemic lockdowns [31]. Forensic psychiatric populations show a likely incidence of evening types [32].

Given that biological rhythms are synchronized to environmental cycles, leaving traces on various aspects of an individual life, it could be seen as a potential tool for retrieving information on the identity of a person in a case of suspicious death. Indeed, many of the key items for human identification, such as age, life span, sex and genetic patterns, are known to be related to clock mechanisms, but the field of chronobiology has not been considered explicitly as tool for forensics yet, probably due to the nonhomogeneous use of terminology.

Therefore, we aim to point out the links between biological rhythms and forensically relevant aspects, including time of death, post-mortem interval (PMI), entomology and surrounding evidence, sex, age, ethnicity, lifestyle factors and developmental features, emphasizing the current common ground of chronobiology and forensics towards an interdisciplinary approach in forensic sciences.

## 2. Circadian Rhythms after Death

### 2.1. Assessment of Time of Death and Post-Mortem Interval

Just as watches stop ticking at a certain point, the molecular feedback loops of biological clocks stop cycling shortly after death due to the processes of autolysis. Although molecular signaling stops after death, gene and protein material can be collected from human remains, representing a snapshot of the molecules that were present as agents of a certain signaling pathway before death. With appropriate collection and isolation protocols, stable molecules can be gathered without signs of post-mortem changes [33,34,35]. Almost all types of human tissue express the key components of the circadian clock where they oscillate, as the so-called peripheral clocks [36]. The nuclear receptor subfamily 1 group D member 1 (REV-ERBα) is an important transcription factor for the core circadian-clock component aryl hydrocarbon receptor nuclear translocator-like protein 1 (*BMAL1*) [37]. In liver, kidney and heart samples taken during autopsies up to 72 h after death from subjects with a known time of death, *REV-ERBα* had a clear peak within an observation time frame of 24 h in all tissue types [38]. The ratio of *REV-ERBα* and its binding partner *BMAL1* (*REV-ERBα:BMAL1*) was suggested as suitable tool to estimate the time of death. In detail, a ratio of >75 indicates a time of death of 0200–0800 h, a ratio of >50 time of death of 0200–0900 h and a ratio of <25 time of death of 1000–2300 h, independent of sex, age, cause of death, PMI and source tissue type [38]. Associations between circadian core clock components and time of death were also found in autopsy samples from the dorsolateral prefrontal cortex [39]. Pineal tissue collected during autopsies, on the other hand, did not display any rhythmic expression of core circadian clock genes or correlation to time of death [40]. The combination of several rhythmic biomarkers can help to estimate trace deposition timing in a forensic case. Melatonin, cortisol, *HSPA1B*, *MKNK2* and *PER3* blood levels can be used together to predict deposition timing in terms of night/early morning, morning/noon and afternoon/evening, based on statistical models [41].

In addition to core clock genes that interact as peripheral clocks in different tissues, there are also genes that are under clock control. These clock-controlled genes follow a rhythmical expression pattern that parallels the circadian period while not influencing the circadian rhythm itself and they are tissue-specific. The expression of miRNAs, for example, is known to be under such circadian clock control [42]. Individuals who died during daytime or night-time have characteristic expression patterns of miRNA sampled from human vitreous humor [43]. In general, the differential expression of miRNAs was found to be a useful tool to determine PMI [44] and wound vitality [45], which could be combined with information on the rhythmical expression to further specify PMI and wound vitality. Cycling gene expression patterns that were associated with time of death were further found in blood samples [41] and samples from cerebellum, nucleus accumbens, anterior cingulate cortex and hippocampus [46,47], but it is not clear if these cycling genes are also under circadian clock control or rather regulated by other factors. Likewise, other publications report on the associations of time of death and a certain daytime in different patient populations [48,49], but these are not supported by any data that could explain these observations.

The chronotype of an individual, which depends on preferred daytimes for body functions and activities, can be assessed by a questionnaire, such as the morningness–eveningness questionnaire (MEQ), developed by Horne and Östberg [50]. Currently, different approaches using molecular analysis for the chronotype were published, which are more precise and bias-free. One of these approaches could be based on the detection of the rs7221412 genotype, a common polymorphism that is located near the core circadian clock gene *PER1*. Interestingly, this *PER1* polymorphism seems to be associated with daytime at time of death as well [51].

The microbiome has been studied in relation to forensic aspects such as human identification, geolocation and PMI [52], and is considered a potential indicator for decomposition [53]. The composition of a microbiome is not only species-specific, but also seems to vary depending on the season. The microbiome composition of buried piglets and soil shifted significantly in the summer, impacting the determination of the PMI [53]. These seasonal varieties of microbiome components were confirmed in another study using a swine and soil model [54].

### 2.2. Assessment of Cause of Death

According to the current statement of the Centers for Disease Control and Prevention, the leading causes of death are heart disease, cancer, COVID-19, accidents, stroke, chronic lower respiratory diseases, Alzheimer’s disease, diabetes, influenza and nephritis. A cause of death implies multiple factors and is therefore probably too complex to be determinable by a sole factor or even a sole signaling pathway, such as the mechanism of the circadian clock system, but there is evidence that diseases that are considered frequent causes of death are associated with disruptions to or changes in biological rhythms [4]. Given that a physiological heartbeat is rhythmical, it seems likely that the cardiovascular system shares common ground with biological rhythms. Indeed, essential cardiovascular tissue functions follow circadian rhythms, which becomes apparent in the daily variations of blood pressure [55], heart rate [56] or the coagulation cascade [57]. The onset of many deadly cardiovascular diseases occurs predominantly in the early morning, thus, particularly in that time of day when physiological cardiovascular functions have their peak activity [58]. A seasonal tendency of myocardial infarction was observed to occur more likely during winter and particularly between 08:00 AM and 12:00 PM [59]. Sudden Infant Death Syndrome (SIDS) is associated with a range of contributing risk factors, but its precise cause is unknown. A circadian character was found with both the occurrence of SIDS as well as the time point when risk factors for SIDS display their effects [60].

Social life, nowadays, disturbs biological rhythms in many ways as does a job that implies shift work. In the 2002 World Health Report by the World Health Organization, shift work is described as a cause for the disruption of circadian rhythms, increasing the risk of cardiovascular diseases. Disrupted circadian rhythms are also found in a variety of other diseases, which is not surprising, given that many regulatory molecules are under clock control. The chronic disruption of circadian rhythms can stimulate cancer development and was listed as an official probable human carcinogenic by the International Agency for Research on Cancer. Since many regulatory molecules involved in tissue homeostasis are under clock control, the disruption of this control creates a favorable environment for cancer development [61]. In general, such disruptions are caused by longer permanent changes of light exposure or food intake [61]. Sirtuin 1 (SIRT1) is a molecular regulator of the circadian clock; thus, SIRT1 downregulation leads to circadian disruption [62]. Such a SIRT1-dependent circadian disruption favors abnormal inflammatory responses in cells from smokers and patients with chronic obstructive pulmonary disease [63].

## 3. Post-Mortem Interval Determination by Biological Rhythms in Forensic Entomology

Arthropods represent valuable forensic evidence as they are often the first witnesses to arrive at a crime scene, settling on human remains and the surrounding. Different insects are particularly helpful in the estimations of a minimum PMI by determining the age and staging of developmental phases in early PMI periods as well as by determining the members of the arthropod compilation in later PMI periods [64]. As in the case of humans, daily insect behavior follows a circadian rhythm that, mainly, times locomotor activity [65] and feeding [66]. Additionally, behavioral output that occurs only once or a few times in an insect’s lifetime, such as development, reproduction and diapause [67,68], displays some circadian character. *Drosophila melanogaster* is one of the most popular model organisms in chronobiology and was originally used to unravel the molecular mechanisms behind circadian rhythms [69,70]. The transcriptional–translational feedback loop in *D. melanogaster* is regulated by *clock*, *cycle*, *period* and *timeless* [71]. In other insects, such as *Lepidoptera*, the constellation of core clock genes is a combination of mammalian and fly key clock genes [72].

Among fly-related species, *Calliphoridae*, *Sarcophagidae*, *Muscidae* and *Piophilidae* are important representatives in forensic entomology [73]. Correlations between the developmental stages and the minimum PMI are well-documented in *Calliphoridae* that frequently populate a body within hours after death [74]. The diapause onset of *Calliphora vicina* orients itself towards length of daily light and dark periods with cycle lengths of 24 h, pointing to a photoperiodic mechanism that could be orchestrated by self-sustained circadian oscillations [75]. In autumn, for example, short days and long nights compose the characteristic photoperiod that, in turn, induces overwintering in larval diapause in *C. vicina* [76]. While photoperiodic conditions impact the induction of diapause, locomotor activity preserves stable circadian periods over the several generations of *C. vicina* with different incidences of diapause [77]. Serotonin regulates the circadian locomotor activity in *C. vicina*, which in turn is dampened, paralleled by increased length periods, after serotonin downregulation by a neurotoxic agent [78]. The scuttle fly *Megaselia scalaris* settles on bodies, no matter if they are buried, exposed, indoor or outdoor. The locomotor behavior of *M. scalaris* shows stable circadian oscillations in both sexes and in the forensically relevant number of laid eggs as well as the timing of oviposition [79]. In the flesh fly *Sarcophaga crassipalpis*, the thermoperiod influences the *period* expression and the timing of adult eclosion in a much stronger way than photoperiodic conditions under artificial conditions in a laboratory [80] as well as in outdoor soil [81]. The cycling of the clock genes *period* and *timeless* are detectable throughout different developmental stages of *S. crassipalpis*, but pause during the early pharate adult stage and continue again in the late pharate adult stage [80]. The critical time frame for the regulation of eclosion onset by *period* signaling occurs 4.5 h before eclosion [82]. The locomotor activity of *S. crassipalpis* is characteristically of a diurnal type, but entrainment patterns to light–dark cycles differ between males and females [83]. Female *S. crassipalpis* generally have higher activity levels with age and are also able to escape temporarily from a typical diurnal behavior, characterized by extended activity in dark phases [83]. Male *S. similis* seem to be less sensitive to light pulses than females [84]. Liver availability is associated with the age at which these behavioral changes occur; otherwise, circadian periods are independent from sex or liver presence [83].

Members of the *Chironomidae* can be used for minimum PMI estimation as well and are of special interest in the case of cadavers from aquatic environments [85,86,87]. The Antarctic midge has a set of core clock genes that are expressed on its head, including *clock*, *period*, *timeless* and *vrille* [88]. Due to the presence of these core clock components, it would be likely to expect a rhythmical expression, but no oscillation could be found after exposing the population to different artificial photoregimes [88]. In midges derived from marine habitats, an adaption of the biological rhythms to local circumstances was described [89]. In addition to *D. melanogaster*, midges of the genus *Clunio* represent an important model organism in chronobiology. The marine midge *Clunio marinus* can be found at rocky coasts where different geographical locations have different tidal regimes, influencing circadian emergence peaks [90]. Circadian as well as circalunar emergence times of different *C. marinus* strains can adapt to local circumstances, associated with splice variant abundance of calcium/calmodulin-dependent kinase II.1 [89]. In addition to circadian rhythms, insects also display circatidal rhythms in the timing of active and inactive phases of locomotor activity, circalunar and circasemilunar rhythms in the photoreception of moon light and circannual rhythms in the entrainment of photoperiodism [71]. Other biological rhythms beyond circadian rhythms have defined length periods, but they do not have a characteristic set of genes that are specific for a respective biological rhythm [71].

In addition to flies, the community of *Hymenoptera* only plays a side role in forensic investigations and is not considered a model in chronobiology [91], yet it can add valuable information to the PMI and decomposition status of a body [92] and is able to express clock genes [93]. *Trichogramma brassicae* express higher mRNA levels of *clock*, *cycle*, *cryptochrome 2*, *period* and *timeout* in asexual strains in comparison with sexual strains, which are disturbed upon infection with *Wolbachia*, a Gram-negative bacterium that uses different insects as host [93]. Genomic database analyses revealed that also the fire ant *Solenopsis invicta* contains the core clock genes *period*, *cycle*, *clock*, *cryptochrome-m*, *timeout*, *vrille*, *par domain protein 1* and *clockwork orange* with a comparable distribution of expression levels during day and night to that of honey bees [94].

## 4. Sexual Dimorphisms of Circadian Rhythms in Human Females and Males

In the course of human life, the chronotype of a person changes influenced by the genetic background, age, sex and environmental factors [95]. On average, female individuals reach the maximum point of lateness in their chronotype at the age of 19.5 years, while males reach their peak of lateness approximately at the age of 21 years [96]. In the age range from 18 to 30 years, male individuals have a predominant preference for a late chronotype [97]. In this range of age, the perception of light, the strongest known zeitgeber, also differs knowingly. In response to blue-enriched light exposure, the perception of light brightness, vigilant attention and sleep physiology differs significantly sex-dependently with males, who show a higher brightness reception and faster reaction times [98]. The sex differences in chronotypes vanish after reaching the age of, approximately, 50 years [95].

The preferences of early and late chronotypes among the sexes is also reflected by the characteristic secretion times of different molecules to some extent. In the dorsolateral prefrontal cortex, the daily variations of clock genes were found relative to time of death [39]. In detail, *PER2* peaked at 10:38 AM, *PER3* peaked at 10:44 AM and *BMAL1* peaked at 09:23 PM with a generally earlier expression timing of all three genes in females compared to males [39]. The sex-related production of the brain-derived neurotrophic factor (BDNF) shows diurnal variations in male plasma with a peak at 08:00 AM and the lowest point at 10:00 PM with significantly lower concentrations than in females [99]. Curiously, BDNF plasma levels did not show any diurnal variations in females [99]. These findings were confirmed in a study with a 30 h constant routine protocol to omit influencing factors from behavior and environment [100]. In a laboratory experiment under constant routine, females had a higher melatonin amplitude and a lower temperature amplitude than males; the sleep timing was the same in both sexes and sleep occurred at a later biological time for females, marked by an earlier circadian rhythm of core body temperature and pineal melatonin secretion relative to sleep time [101].

The mechanisms that regulate sex-dependent biological rhythms are not elucidated yet, but there are inital findings that demonstrate a genetic contribution to differences between male and female individuals and circadian clock components. Cardiovascular diseases are known to have a different incidence in males and females. In patients who had a myocardial infarction, males had a higher minor allele frequency of the *CLOCK* polymorphism rs11932595 than females [102]. The chromosomal locus that includes the *CLOCK* gene is further linked to opioid dependence in a sex-dependent manner [103].

## 5. Age Determination by Biological Rhythms

An individual chronotype changes over the lifetime of a human being. In childhood, most individuals display early chronotypes, which become later when entering puberty and continue to delay throughout adolescence, until the lateness of a chronotype reaches a peak at 19 or 21 years in human males and females, respectively. After reaching this turning point, chronotypes become earlier again [95].

**Figure 1:**
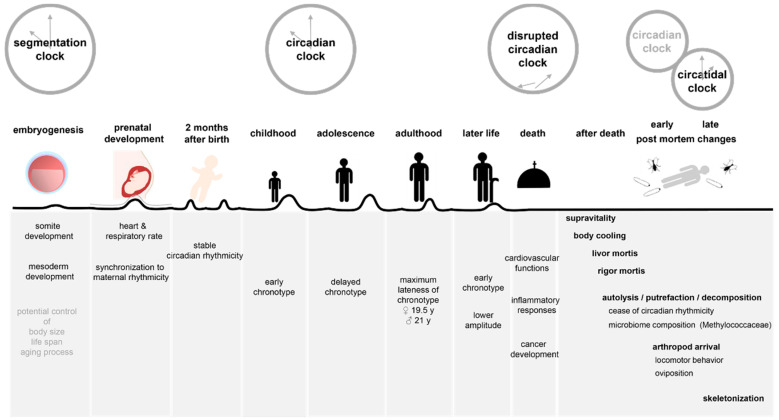
Biological rhythms of life and death. This figure shows how different clock rhythms are involved in the different stages of life as well as the early and late post-mortem changes after death.

Circadian rhythmicity accompanies humans throughout their entire life (Figure 1), already starting in utero. In parallel to the prenatal development of the suprachiasmatic nucleus that hosts the central circadian clock, the circadian clock starts to form in the fetus [104]. In the fetal stage, rhythmicity of, e.g., the heart and respiratory rates are in phase with the maternal rhythmicity [105]. After birth, infants already display certain morningness–eveningness activities during the first week, while establishing a stable circadian rhythmicity takes approximately two months [104]. Likewise, disturbances of the circadian rhythm, as it is the case with shift work, are transferred from mother to child, exhibiting an increased risk of metabolic disorders in their offspring [106].

The process of aging is mediated by a complex interplay of signaling pathways, of which those that are responsible for nutrient sensing and metabolism are particularly important, including the SIRT and mTOR complexes. Some of the essential nutrient-sensing and metabolic pathways are in turn involved in the molecular feedback loop downstream of the core circadian clock mechanism and most of the molecular mediators of these pathways show circadian oscillations in different organs [107], which points to a close connection between the processes of aging and circadian clock mechanisms. In elderly populations, chronotypes become earlier again and sex-specific differences vanish. Older adults complaining of insomnia show a disturbed synchronization of circadian rhythms compared to younger adults [108], while healthy old adults generally do not show any signs of circadian rhythm dampening or disruption [109].

### 5.1. The Epigenetic Clock

Age estimation for forensic purposes is based on a variety of methods that are often used in combination. Medical images of teeth or bones and their morphological assessment constitute a traditional approach to estimate chronological age. As the morphological development of denture and skeleton is marked by high differentiation during childhood and adolescence and decreases afterwards, these approaches are more reliable on specimens of a respective age range [110]. In adulthood, i.e., an approximate age range of 20 to 50 years, there are neither age-associated dental or skeletal morphological changes nor is there a characteristic circadian rhythmicity for these decades of life. Epigenetic clocks could fill this gap and provide a consistent source for chronological age estimations across all ages. Thereby, the CpG methylation levels of age-associated genes can be used to predict the age within a variance of approximately 3 years in blood [111], buccal cells and bone [112]. A similar approach used a combination of methylation markers of five different genes to calculate the age from blood samples based on machine learning, reporting a similar prediction precision with difficulties in samples of older adults [113]. Environmental changes leave their traces on aging, epigenetics and circadian rhythms. Although the epigenetic clock is not meant as a biological rhythm, such as the circadian clock, epigenetic modifications share mutual regulatory mechanisms with circadian rhythms and aging [114]. Effects of environmental exposure are of particular importance in forensic investigations and can be taken into account when predicting age with the help of DNA methylation patterns [115].

The epigenetic clock comprises the assessment of DNA methylation patterns in biomarkers of aging and the use of mathematical models to predict epigenetic and chronological age. The molecular mechanisms of the circadian clock highly rely on epigenetic regulation at different levels to enable rhythmic transcription, establish chromatin loops and distribute circadian clock genes correctly within the cell nucleus [116]. Mammalian animal models revealed that not only DNA methylation, but also histone posttranslational modifications play a role in the context of circadian clock mechanisms. In the central circadian clock, the phosphorylation of histone H3 at Serine 10 is highly photosensitive in circadian clock cells of the SCN, providing dynamic chromatin remodeling in adaption to cyclic light periods in the environment [117]. Additionally, in peripheral tissues, a circadian rhythmicity in cytosine modifications became evident [118]. In forensic autopsy samples from blood, heart, lung, liver, kidney and brain, the promotors of *PER1*, *CRY2*, *BMAL1*, *CLOCK* and *CK1e* were found to be unmethylated in all sample types and the promotors of *PER2*, *PER3*, *CRY1* and *TIM* were partially methylated [119]. The found methylation patterns had inter- and intraindividual variations and they were further altered in cases with known methamphetamine exposition [119]. The promotor regions of *Per1*, *Per2* and *Cry1* are marked by rhythmic H3 acetylation as well as RNA polyermase II binding, which also corresponds to the rhythmic mRNA expression [120]. Together with the analysis of histone posttranslational modifications, an epigenetic-circadian landscape in the murine liver could be defined, characterized by three main cycling phases of circadian transcription: a poised state, a state of transcriptional activation and a state of repression [121].

The prediction of age based on the epigenetic clock can be divided into the determination of epigenetic and chronological age. If the epigenetic age of a person, determined by the DNA methylation pattern status, is higher than the actual chronological age of the individual, the status of this individual is referred to as an accelerated epigenetic age. Epigenetic acceleration can offer hints to different personal identifiers, such as general body fitness or ethnical background. Developmental characteristics such as the amount of fat mass or body height are related to such epigenetic age acceleration [122]. The rates of epigenetic acceleration measured in blood, relative to the blood cell counts differ between ethnicities. In a declining order, Hispanics and Tsimane Amerindians have the highest epigenetic acceleration rates, followed by Caucasians and African Americans [123]. It is known that socio-physiological environmental factors can impact the chronotype [124], but the relation between circadian rhythms, ethnical background and the epigenetic clock has not been clarified to date.

### 5.2. Biological Rhythms in Development

As development is timed by a clock and development occurs in adaption to environmental conditions, it suggests a certain connection between biological clock mechanisms and individual features of the human body that is characteristic for a certain population, culture or social environment. The segmentation clock regulates the timing of somite development, which occurs rhythmically during embryogenesis [125]. By that, the oscillations of the segmentation clock contribute to the development and patterning of the mesoderm. Originally, the segmentation clock was discovered in chick embryos [126] and was later studied in different vertebrate models [127] and arthropods [128]. The use of pluripotent stem cells enabled the study of the human segmentation clock in more detail [129,130]. The human segmentation clock displays a period of approximately 5 h and is driven by a total of 219 clock genes that are involved in Notch, ERK/MAPK, Wnt/β-catenin, PI3K/AKT, protein kinase A, Hippo, TGF-β, ephrin receptor, BRD4, ID2, HDAC, YAP1 and clock signaling pathways [129]. Interestingly, the periods in human segmentation clocks are almost as doubly long as in mice [127]. The protein Hes7 plays an essential role in the mechanisms of the segmentation clock and was found to be degraded slower in human cells than in murine cells [127], but the exact regulatory mechanisms that determine the speed of segmentation clock oscillations is not completely clarified to date. This finding is supported by the correlation between the rate of proteome turnover, metabolic demands and life span in different species [131]. Taken together, these cellular clocks could be responsible for the control of body size, life span and the aging process of an individual [132], all of which could be of immense interest for forensics, but this remains to be studied in the future.

## 6. Discussion

This narrative review intends to link the current knowledge between biological clocks and forensically relevant aspects, such as the PMI, causes of death, forensic entomology, sexual dimorphisms, age estimation and the developmental characteristics of an individual. The largest part of the literature on biological clocks does not explicitly mention its relevance to forensics and has therefore also not been evident to a wide extent until now. Both disciplines have their own technical terminology, which probably masks the otherwise evident common ground of both fields.

Nevertheless, the presented data provide some basis for the specialty of forensic chronobiology, but significantly more research and development are required until this knowledge can be transferred to real-life applications in forensic investigations. Findings at molecular levels still have a rather descriptive character or deliver masses of complex data that cannot be evaluated manually anymore. On the way to the practical implementation of this knowledge, mathematical modeling [133] and integration into machine learning approaches [134] will be required to understand relevant associations between biological rhythms, factors from the environment and to finally extract forensically relevant information. Algorithm-based approaches will enable to connect morphological and functional data to omit their respective limitations, one of these limitations being the need for reference material that is required but hard to obtain in many different forensic methods.

Although this narrative review shows that the circadian clock is the objective of several forensically relevant studies, most of them have a rather descriptive character, reporting observations that are related to the circadian clock, but do not further investigate the underlying circadian mechanisms behind these observations. Thus, further mechanistic studies will strongly support the developments in forensic chronobiology. Future studies will also profit from research in human cells, clinical studies or autopsy specimens rather than animal models. It should be kept in mind that the biological rhythms of animals, such as rodents, are very different from those in humans and, while they can be of value to broaden biological understanding, they are not transferrable to forensic sciences, which requires human-based information. Research in aging is a representative example where life span and metabolism between humans and rodents are not comparable and categorizing animals as young and old in most cases confounds biological life span and life expectancy under laboratory conditions.

This review demonstrates that findings in chronobiology that were directly linked to a forensically relevant aspect were all connected to circadian rhythmicity (Table 1), while all other findings presented in this review either lack assessment of a forensic output or the analysis of biomarkers of the biological rhythms within one study. Thus, this review attempted to emphasize that the building blocks for both fields are already present, but they need to connected in future studies. To date, the role of circannual, circalunar and circatidal rhythms has not been studied in humans even though it was hypothesized that at least circannual and circatidal rhythms could be associated with reproduction and mental health [135]. Additionally, biological rhythms are not necessarily separately acting mechanisms, but their ways of interactions and influences on each other have to be clarified further. Recently, a study revealed that moon light also impacts the timing of daily behavior in addition to its known impact on the timing of monthly behavior in *P. dumerilii* [136]. Thus, future studies need to determine the presence and role of circannual and circalunar rhythms in humans in order to evaluate potential relevance to the PMI or age determination in later studies, together with the already existing knowledge of individual chronotypes and circadian rhythms. As humans nowadays live far from natural conditions, social time, in addition to Earth cycles and clock time, should be studied in association with chronotype and biological rhythms together with forensically relevant aspects.

Arthropods, on the other hand, are already known to display behaviors that underlie circadian, circalunar and circatidal timing. Here, translational research will be required to transfer this knowledge to practical applications in forensic entomology.

Taken together, broadening the knowledge on biological rhythms in forensically relevant terms is worth of profound research as it bears high potential to bring forensic sciences to a next level, keeping up with rapid developments in lifestyle and criminality.

## Figures and Tables

**Table 1:** This table shows an overview of findings that are both linked to a forensically relevant aspect as well as biomarkers of circadian rhythmicity.

Forensic Output	Biomarkers	Tissue Source	Reference
time of death	*REV-ERBα:BMAL1*	liver, kidney, heart	Kimura A. et al., 2011 [38]
	*PER2*, *PER3*, *BMAL1*	dorsolateral prefrontal cortex	Lim A. S. P. et al., 2013 [39]
daytime of death	melatonin, cortisol, *HSPA1B*, *MKNK2*, *PER3*	blood	Lech K. et al., 2016 [41]
	mir-34c, mir-541, mir-888, mir-484, mir-142-5p	vitreous humor	Odriozola A. et al., 2013 [43]
	*PER1* polymorphism rs7221412	cerebral cortex, peripheral blood mononuclear cells, CD14+CD16− monocytes	Lim A. S. P. et al., 2012 [51]
post-mortem interval	Methylococcaceae	buried piglets and soil	Olakanye A. O. et al., 2017 [53]
	locomotor behavior, oviposition	*Megaselia scalaris*	Bostock E. et al., 2017 [79]
sex dimorphism	*PER2*, *PER3*, *BMAL1* peak time	dorsolateral prefrontal cortex	Lim A. S. P. et al., 2013 [39]
	*CLOCK* polymorphism rs11932595	peripheral blood lymphocytes	Škrlec I. et al., 2021 [102]
characteristic age span	chronotype	NA	Roenneberg T. et al., 2007 [95]
cause of death	methylation status of *PER1-3*, *CRY1-2*, *BMAL1*, *CLOCK*, *CK1e*, *TIM* promotors	blood, heart, lung, liver, kidney, brain	Nakatome M. et al., 2011 [119]
	myocardial infarction daytime	NA	Sakelliadis E. I. et al., 2021 [59]

## Data Availability

Not applicable.
